# Positive and Negative Affect Change among People Living with HIV: a One-Year Prospective Study

**DOI:** 10.1007/s12529-018-9741-0

**Published:** 2018-08-10

**Authors:** Marcin Rzeszutek, Ewa Gruszczyńska

**Affiliations:** 1 Faculty of Psychology, University of Warsaw, Stawki 5/7, 00-183 Warsaw, Poland; 2 Faculty of Psychology, University of Social Sciences and Humanities, Chodakowska 19/31, 03-815 Warsaw, Poland

**Keywords:** HIV, Affect, Gender, CD4 count

## Abstract

**Purpose:**

The aim of this study was to investigate the heterogeneity of changes in affective states, i.e., positive (PA) and negative (NA) affect, as well as the sociodemographic and clinical covariates of these changes among people living with HIV (PLWH) in a 1-year prospective study.

**Method:**

Participants were 141 ambulatory patients (15% female) with a confirmed diagnosis of HIV infection who were undergoing antiretroviral treatment. Their affective states were assessed three times, with 6-month intervals, using the positive and negative general affect scale (PANAS-X). Sociodemographic (gender, age, relationship status, education, employment) and clinical variables (CD4 count assessed via self-report, HIV/AIDS status, time since HIV diagnosis and antiretroviral treatment duration) were also obtained.

**Results:**

Heterogeneity of changes was present only for NA, whereas PA decreased gradually in the whole sample. Time since diagnosis was unrelated to baseline affect levels as well as affect level changes. Additionally, the trajectories of NA and PA were independent of each other. The significant correlates of trajectories were gender and CD4 counts, both baseline CD4 levels and CD4 changes.

**Conclusion:**

This study adds to the literature by describing affect changes among PLWH and identifying potential correlates of these changes, particularly CD4 count and gender. As such, these findings point to the potential clinical significance of further research on the roles of these variables.

## Introduction

There is a great debate on the process of the adaptation of subjective well-being (SWB) as result of experiencing various stressful life events [1–3], including specifically the case of struggling with chronic illness [4–6]. A considerable amount of research shows evidence for the ‘hedonic treadmill’ model [7], i.e. ‘stability despite loss’ of SWB in reaction to various life stressors [8, 9]. However, the aforementioned studies dealt only with one cognitive aspect of SWB, namely, life satisfaction, which is defined as a relatively stable, global evaluation of a person’s life [1]. Much less investigated, with a plethora of conflicting results, is the adaptation of the affective components of SWB in the wake of chronic stressful situations, usually operationalised as the presence of positive (PA) and negative (NA) affect [10]. On one hand, some studies indicate a relative stability of NA compared to PA, as the former component of affective well-being is rooted more in personality than in situational factors [11]. Conversely, Mroczek and Almeida [12] found significant within-subject fluctuations of NA in a daily diary study, which may indicate that assessment of NA shows different results in short and long time periods. On the other hand, there are authors who have found that both PA and NA are equally prone to context-related features [13].

According to Diener et al. [1], these mixed results may be attributed to the fact that the term ‘adaptation’, as described above, is sometimes improperly used as a synonym of similar concepts, such as coping with stress. More precisely, adaptation is a broader concept than coping, as it refers to one’s global reaction to a changing environment and may not always be associated with intentional self-regulation. In addition, Diener, Lucas and Scollon [14] noted significant individual differences within the set point of SWB and the time required to return to an individual set point, as well as different trajectories of changes in various components of SWB, i.e., life satisfaction vs. affective components. Individual differences in the adaptation of SWB are especially visible in cases of stress caused by chronic diseases, in which this adaptation does not indicate, as previously mentioned, stability despite loss but consists of temporal, intraindividual variations in SWB, depending on the stage of the illness as well as various socio-medical resources [6]. One such illness that requires complex social adaptation and is related to dynamic changes in SWB, especially its affective components, is HIV/AIDS [15, 16].

Remarkable progress in the treatment of HIV has caused a change in the nature of HIV/AIDS from terminal to a chronic, manageable illness [17]. Nevertheless, it is symptomatic that people living with HIV (PLWH) declare consistently lower levels of well-being, not only in comparison with the general population [18] but also in respect to other chronic diseases [19]. There is an immense body of literature pointing to the existence of intense and often unpredictable HIV-related distress among PLWH (e.g. [20–23]), which, currently, is more related to psychosocial factors than to medical outcomes, considering the abovementioned chronic and manageable nature of this disease [24]. The aforementioned distress is clearly present among newly diagnosed PLWH [25]. However, some PLWH continue to suffer from high levels of depression as well as negative affect many years after the diagnosis [26], which may be related to poorer health outcomes [20], risky sexual behaviours [27] and poor adherence to treatment [25]. Nevertheless, a longer time since being diagnosed with HIV may facilitate well-being adaptation, especially if the disease is well-controlled through antiretroviral treatment [28]. Conversely, a few authors have also observed relatively stable levels of positive affect among PLWH several years after the diagnosis, which has been linked to slower HIV progression [29], better adherence to treatment [30] and decreased risk of mortality [31]. Importantly, the aforementioned effects of NA and PA on various aspects of the functioning of PLWH were found to be mutually independent [16].

## Current Study

Although many studies exist that have assessed affective well-being among PLWH, the majority of them, presented above, were either conducted in a cross-sectional framework or were based only on the analysis of changes in the mean scores of the study variables. In other words, they presented general trends for the whole study sample and neglected the unique trajectories of changes among particular subgroups of participants with different socio-medical characteristics. Thus, little is known about the heterogeneity of changes in affective states (NA and PA) among this patient group. Therefore, the novelty of this study is that we used latent class growth modelling (LCGM) analysis [32] to identify subgroups of participants on the basis of their trajectories of changes in NA and PA, which are further expected to differ with respect to various socio-medical variables.

Particularly, we formulated three study hypotheses:

1. We expected that changes in PA and NA over time would be negatively related to HIV duration. Specifically, HIV duration should be positively related to PA and negatively related to NA at baseline and negatively related to affect changes, i.e. affect changes should decline with increasing time since HIV diagnosis.
2. We expected that both the starting points and the amount of changes in PA and NA would be mutually independent.
3. We expected heterogeneity of changes in PA and NA, i.e. different classes of trajectories of PA and NA with respect to different socio-medical correlates (see Table 1) of trajectory membership.

**Table 1:** Sociodemographic and HIV-related characteristics of the studied sample (*N* = 141)

Variable	Number (%)
Gender	
Male	120 (85.1%)
Female	21 (14.9%)
Age in years	
M ± SD (range)	40.18 ± 10.24 (19–76)
Stable relationship status	
Yes	84 (59.6%)
No	57 (40.4%)
Education	
Elementary/secondary	61 (43.3%)
University degree	80 (56.7%)
Employment	
Full employment	99 (70.2%)
Unemployment/retirement	42 (29.2%)
HIV/AIDS status	
HIV+ only	120 (85.1%)
HIV/AIDS	21 (14.9%)
HIV infection duration in years	
M ± SD (range)	7.34 ± 6.20 (1–30)
Antiretroviral treatment (ART) duration in years	
M ± SD (range)	5.67 ± 5.10 (1–23)
CD4 count (assessed via self-report)	
T1 = M ± SD (Range)	609.57 ± 240.90 (200–2000)
T3 = M ± SD (Range)	597.27 ± 207.75 (145–1255)
T3–T1 residuals = M ± SD (Range)	0 ± 204.77 (− 452.22–649.08)

*M* mean; *SD* standard deviation; *T1*, *T3* measurement points: *T1* baseline, *T3* 1 year after baseline

## Method

### Participants and Procedure

Participants were patients of the outpatient clinic in a hospital of infectious diseases. After informed consent was obtained, the participants filled out a paper-and-pencil version of the measures and participated in the study voluntarily, as there was no remuneration for participation. The study’s eligibility criteria included the following: aged 18 years or older, medically diagnosed as HIV-positive and currently receiving medical care from the clinic where the study was performed. The exclusion criteria included HIV-related cognitive disorders diagnosed by medical doctors working at the hospital. This study was approved by the local ethics committee.

The first assessment was performed during June and July 2016. A total of 141 patients agreed to participate in the study and provided their contact details (i.e. phone number and/or e-mail address). The second assessment was conducted during January and February 2017. Out of 141 participants from the first assessment, 113 took part in the second assessment. The final assessment was completed during May and June 2017, with 82 participants remaining. Table 1 presents the socio-medical characteristics of the study sample.

## Measures

Positive and negative affect was assessed with 20 descriptions of emotions, 10 for positive affect (e.g. ‘proud’, ‘excited’) and 10 for negative affect (e.g. ‘nervous, ‘upset’), from the PANAS-X by Watson and Clark [33]. Participants rated their general affective state during the previous month on a 5-point response scale from 1—*not at all* to 5—*strongly.* Cronbach’s alpha coefficients for the PA scale were .91, .94 and .89, and for NA scale, −.92, .91 and .91, for the three measurement points, respectively.

## Data Analysis

**Fig. 1:**
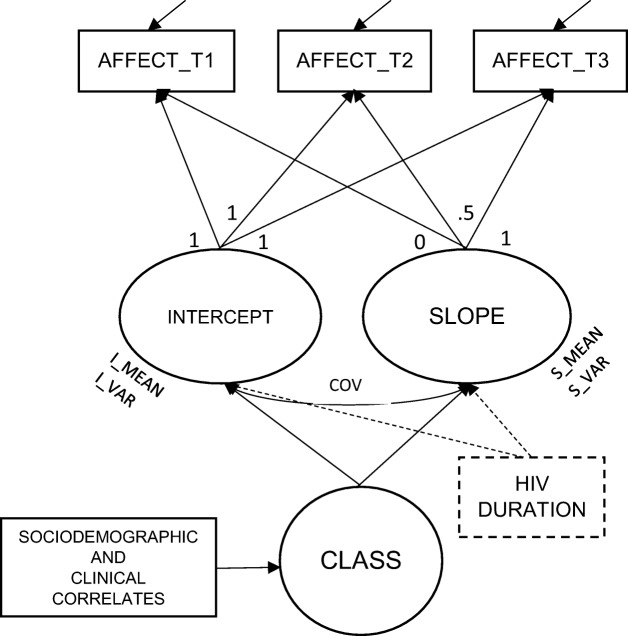
A latent trajectory model with three observed repeated measures of affect (AFFECT_T1, T2 and T3); two growth parameters (INTERCEPT and SLOPE) and possible heterogenity of trajectories (CLASS) with covariates. I_MEAN, S_MEAN—an average values of affect baseline level and change during time of the study, respectively; I_VAR, S_VAR—variance of intercept and slope, i.e. interindividual variability in affect starting point and change, respectively; COV = covariance between intercept and slope. Error variances symbolised with arrows pointing to AFFECT variables. HIV DURATION—time-invariant antecedent in conditional model (see details in the text)

Latent growth curve analysis (LGC, [34]) was used to verify hypothesis 1 and 2, and a latent class growth curve (LCGC, [32]) was performed to verify hypothesis 3. In the linear LGC, changes in repeated measures over time are expressed with two factors: the intercept (initial status) and the slope (growth rate) (see Fig. 1). Since the measurement intervals were equal, time was coded as 0 for the first measurement (T1), 0.5 for the next wave 6 months later (T2) and 1 for the last wave, around 12 months after the first measurement (T3). Thus, the intercept mean can be interpreted as an average affect value for the whole sample at the beginning of the study, whereas the slope mean is an average change between T3 and T1 for the whole sample. Additionally, variations around the mean values of the intercept and slope were assessed to verify if the sample was homo- or heterogenous in terms of individual starting points and/or growth rates. This model, called unconditional, was established for PA and NA separately. Next, in a conditional model, HIV duration was assumed to affect both the intercept and slope of PA and NA. Finally, a model where PA and NA were tested together in a bivariate latent growth curve model (called also a parallel process model [35]) was used to examine whether their starting points and growth rates were related to each other. To assess the goodness of fit of the above described models, chi-square tests (*χ*^2^), the Tucker-Lewis Index (TLI), a comparative fit index (CFI) and the root mean square error of approximation (RMSEA) with a 90% confidence interval were used [36]. The model was assumed to fit the data reasonably well when the RMSEA was below .08 and both TLI and CFI were above .90. This part of analysis was completed using Mplus version 8 [37].

To examine the heterogeneity of the trajectories, an LCGC was applied. The number of classes was distinguished on the basis of four categories of indices: (1) the Bayesian information criteria (BIC), the Akaike’s information criterion (AIC) and the sample size-adjusted BIC (SABIC); (2) the bootstrap likelihood ratio test (BLRT) to compare model with k class and k-1 class; (3) entropy values [38]; and (4) substantive criteria [32], just like a size of the smallest class.

Sociodemographic and HIV-related variables, such as gender, age, education, employment, HIV duration, antiretroviral treatment (ARV) duration, AIDS and self-reported CD4 count at baseline were tested as potential time-invariant covariates of class membership. Also, the CD4 change over the length of the study (residuals of T3 on T1) was included in the model as a possible time-variant covariate to verify if the change in CD4 count was related to any specific affect trajectory. Although other variables can be time-variant as well (e.g. relationship or employment status), there was not enough overtime variability in our sample to apply such an approach (for instance, only 7 of 141 participants reported a change in relationship status between T1 and T3). All covariates were examined in a bias-adjusted three-step approach [39] that corrected for classification errors with the maximum likelihood (ML) method. For this part of the analysis, Latent GOLD 5.1 [39] was used. Descriptive statistics, missing data analysis and imputation were performed with IBM SPSS Statistics version 24 [40].

## Results

### Missing Data Treatment and Descriptive Statistics

Since between the first and third measurements we noted a dropout of 42%, we examined if the sample attrition was systematic in any specific way [41]. Non-completers did not differ significantly from completers in any of the studied variables, including sociodemographic characteristics. Additionally, Little’s test results (*χ*^2^ = 17.83, *df* = 14, *p* = .22) indicated that the missingness could be treated as random. Thus, based on current recommendations, the missing data were imputed using predictive mean matching [42] and further analysis were performed for *N* = 141. Table 2 illustrates the descriptive statistics for the repeated assessments of affect. As seen in this table, at each measurement point, the distributions were not differing significantly from normal.

**Table 2:** Descriptive statistics of repeated measures of positive and negative affect

	Positive affect			Negative affect		
	T1	T2	T3	T1	T2	T3
M	3.43	3.39	3.30	2.31	2.08	2.20
SD	0.65	0.78	0.68	0.93	0.81	0.93
Kurtosis	− 0.08	− 0.54	− 0.55	− 0.76	0.52	− 0.27
Skewness	− 0.35	− 0.09	0.17	0.48	0.88	0.86
Minimum	1.6	1.4	1.7	1	1	1
Maximum	4.7	5	4.6	4.5	5	4.6

*M* mean, *SD* standard deviation; *T1, 2, 3* measurement points: *T1* baseline, *T2* 6 months after baseline, *T3* 1 year after baseline

- Hypothesis 1: NA and PA changes over time and their relationship with HIV duration

We began by verifying unconditional models of changes in PA and NA separately (univariate latent growth models). The results are presented in Table 3. For PA, a significant decrease over time was observed, and the sample did not differ significantly in this regard (variance around slope was equal zero). A different pattern was noted for NA. The mean slope for the sample suggested no significant change during the study, but the significant variance around the slope indicated possible heterogeneity of the changes, which were examined further in the latent class growth curve analysis. Additionally, a significant covariance between the intercept and slope (− .37, *p* = .005) demonstrated that higher baseline values of NA were related to lower slope values, thus to a bigger decline over time. However, the model for NA despite an insignificant *χ*^2^ value had other goodness of fit parameters slightly below satisfactory levels; consequently, these results should be interpreted with caution.

In the next step, we tested whether HIV duration was a significant predictor of either baseline affect or its changes over 1 year. In both conditional models, i.e. for PA and NA, time since diagnosis with HIV was unrelated to affect values at the first measurement point as well as affect changes over 1 year. For NA, adding time since diagnosis to the model worsened the goodness of fit far below a reasonable level.

**Table. 3:** Unconditional and conditional model of latent growth curve for positive and negative affect

Model	Growth parameters				Goodness of fit indices			
	Intercept		Slope		*χ* ^2^ (*df*)	TLI	CFI	RMSEA (90% CI)
	M (SE)	Var (SE)	M (SE)	Var (SE)				
Unconditional								
PA	3.43*** (.05)	.20*** (.04)	− 0.13*** (.06)	0^a^	2.79 (3)	1	1	0 (0, .14)
NA	2.19*** (.05)	.41*** (.11)	0^b^	.68*** (.22)	8.23 (4)	.86	.89	.09 (0, .17)
Conditional								
	Intercept on HIV duration		Slope on HIV duration					
PA	.009 (.009)		− .002 (.009)		3.65 (4)	1	1	0 (0, .12)
NA	0 (.01)		.002 (.01)		12.81** (5)	.61	.67	.11 (.03, .18)

*PA* positive affect; *NA* negative affect; *M* mean, *Var* variance, *SE* standard error; *χ*^2^ (*df*) chi-square test with degrees of freedom, *TLI* Tucker-Lewis index, *CFI* comparative fit index, *RMSEA* root mean square error of approximation, *90% CI* 90% confidence interval of RMSEA

*p < .05; ** *p* < .01; *** *p* < .001

^a^Non-significant negative slope variance fixed to zero

^b^Mean slope fixed to zero and error variances of repeated measures set to be equal

- Hypothesis 2: Independence of PA and NA starting points and changes

In bivariate latent growth model of PA and NA (*χ*^2^ = 23.31, *df* = 14, *p* = .06, TLI = .89, CFI = .90, RMSEA = .07, 90% CI (0, .12)), the covariances between intercepts (.006, *ns*) as well as between slopes (0) were insignificant. Since the slope variance for PA was set to 0, all continuous latent variable covariances involving this parameter were fixed at 0. Thus, as assumed, NA and PA were unrelated at the starting point, and so were their changes over the length of the study.

- Hypothesis 3: Heterogeneity of NA changes and its covariates

For PA, the variance around slope was insignificant, indicating homogenous changes in the sample. Thus, latent classes of change were examined only for NA. Table 4 represents the goodness of fit characteristics for models with different numbers of classes. The lowest value of BIC was at a 3-class solution. Additionally, number of the smallest class suggested a solution with no less than three classes. Other indicators showed a clear improvement between 1- and 2-class solutions, with more ambiguous values regarding further class differentiation. However, as BIC can be regarded as the most valid indicator of all information criteria considered in the study, especially for the smaller sample size [38], we decided on a 3-class model.

**Table 4:** Parameters of models with different number of latent classes of negative affect trajectories (*N* = 141)

Model	BIC	AIC	SABIC	Number of parameters	Entropy	BLRT		Smallest class	
						value	*p*	% of *N*	frequency
1-Class	1116.17	1107.31	1106.67	3					
2-Class	1044.36	1023.71	1022.20	7	0.81	91.61	< .001	26.95	38
3-Class	1048.68	1016.25	1013.88	11	0.71	15.46	.010	20.57	29
4-Class	1051.85	1007.62	1004.39	15	0.72	16.63	.012	3.55	5
5-Class	1059.54	1003.51	999.42	19	0.72	12.11	.032	3.55	5

*BIC* Bayesian information criterion; *AIC* Akaike’s information criterion; *SABIC* sample size-adjusted BIC; *BLRT* bootstrap likelihood ratio test

**Fig. 2:**
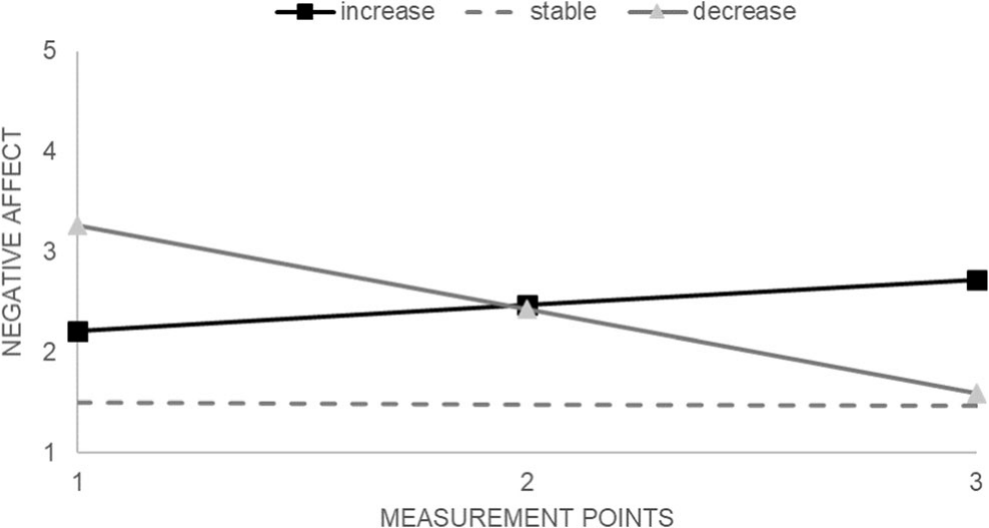
Three trajectories of negative affect: results of latent class growth curve analysis

This solution is presented in Fig. 2. The most frequent trajectory of NA changes was an increasing trajectory (51% of the sample; intercept = 2.21, *z* = 18.25, *p* < .001; slope = 0.50, z = 2.09, *p* = .034), followed by a stable trajectory (27%; intercept = 1.50, *z* = 26.66, *p* < .001; slope = − 0.03, *z* = − 0.41, *ns*) and a decreasing trajectory (22%; intercept = 3.26, *z* = 15.87, *p* < .001; slope = − 1.68, *z* = − 5.14, *p* < .001).

A 3-step approach with adjustments for classification errors and class-independent error variances was applied to establish class membership covariates. Among the tested variables, only gender (Wald = 9119.06, *p* < .001); self-reported baseline CD4 count (Wald = 7.64, *p* = .02); and CD4 change (T3–T1 residuals, Wald = 8.30, *p* = .02) were significant predictors of class membership. Namely, there was an overrepresentation of women in the increasing trajectory class: 95% of the female participants were members of this class, whereas none of the female participants were represented in the stable trajectory class (see Table 5). For baseline CD4 count, pairwise comparisons revealed that members of the stable trajectory had the highest CD4 values, with no significant differences between the increasing and decreasing trajectories. Interestingly, these classes differed significantly in CD4 changes: only in the NA increasing trajectory was a CD4 decrease noted. In other words, it was not the baseline CD4 counts that were distinctive of the increasing or decreasing trajectory classes, but the direction of the CD4 change.

**Table 5:** Significant covariates of latent classes of negative affect trajectories

Covariates	Negative affect trajectories		
	Increase (1) *n* = 73	Stability (2) *n* = 39	Decrease (3) *n* = 29
Gender			
Men	53	39	28
Women	20	0	1
Cross-tabulation comparison	*χ*^2^ = 18.83, *df* = 2, *p* < .001		
CD4	M (SD)	M (SD)	M (SD)
Baseline count	595.53 (214.74)	689.62 (302.56)	542.28 (181.80)
T3–T1 change	− 78.02 (168.70)	81.34 (214.84)	86.89 (202.34)
Pairwise comparisons	1 vs 2	1 vs 3	2 vs 3
CD4 baseline count	4.71*	0.79	6.09**
CD4 T3–T1 change	7.51**	7.72**	0.15

*M* mean, *SD* standard deviation; *T1*, *T3* measurement points: *T1* baseline, *T3* 1 year after baseline; for pairwise comparisons Wald values with *df* = 1

*p < .05; **p < .01; ***p < .001

## Discussion

Three hypotheses regarding affect changes among PLWH were tested in our study. The first hypothesis was not confirmed, as neither the baseline levels of PA and NA nor their changes were related to HIV infection duration. The abovementioned finding is somewhat contrary to other studies on the adaptation of affective well-being in case of stress induced by chronic illness [6, 43]. More specifically, Schilling & Wahl [6] found in a group of patients suffering from macular degeneration that changes in PA were nonlinearly associated with the duration of this disease, but the NA level was stable, which is in line with classic theories on the dispositional nature of NA [44]. In contrast, the results obtained in our sample may point to an affective adaptation to HIV infection, as our participants had been diagnosed with HIV at least for 1 year. Thus, this finding corresponds to the previously mentioned hedonic treadmill model [7], i.e. stability despite loss of SWB in reaction to various life stressors [9]. Additionally, it should be noted that PA declined gradually in the whole sample throughout the study year, and we did not observe any interindividual variabilities in this trend. This topic requires further empirical exploration, especially in the context of HIV-related depression. More specifically, to date, the majority of research on depression among PLWH has been concentrated mainly on the NA experience, i.e. various aspects of HIV-related distress [26, 45], and has minimised the issue of PA, which is associated with various health-related benefits in this patient group [15]. Furthermore, when discussing the potential differential contribution of affect dimensions, an interesting hypothesis of health-related benefits of mixed emotions should be mentioned [46], especially as this advantageous effect has been very recently observed also among PLWH. Batchelder et al. [47] have shown that experiencing positive emotions along with negative self-conscious emotions evoked by self-reflection (e.g. shame, guilt and embarrassment) may be related to reducing several HIV-related maladaptive behaviours, including HIV transmission.

In line with the second hypothesis, we found that both the starting points and the amount of change in PA and NA were mutually independent. This corresponds with previous studies pointing to similar trends with respect to these affect valences [48] that are usually interpreted as the result of different neurobiological underpinnings of PA and NA [49]. In addition, diverse but complementary functions of PA and NA in the process of adaptation to stressful situations have been mentioned [50]. Namely, whereas NA causes a narrowing of cognitive-behavioural responses to stressors, PA broadens these aforementioned responses, simultaneously building up an individual’s resources, which, in turn, may regulate the level of NA. The independence of PA and NA has also been found in several studies conducted on PLWH, yet the heterogeneity of trajectories was not tested in these studies [31, 51]. Therefore, our findings fill the research gap in this area and may suggest that in HIV counselling, PA and NA should be treated as separate and equally important dimensions, and in this manner, they should be assessed and monitored, which has also been mentioned in recent studies [16].

The last hypothesis was partially supported, as heterogeneity of changes was noted only with regard to NA but not with respect to PA. Regarding NA, three classes of changes were observed, i.e. increasing, stable and decreasing trajectories, of which the NA increasing trajectory was the most salient class. Thus, the results obtained in our study may reveal the uniqueness of affective processes among PLWH. Namely, our findings can be interpreted in light of the considerable amount of studies pointing to the chronic presence of negative affectivity, especially depression, among PLWH, even many years after the HIV diagnosis [20, 26, 52]. In particular, PLWH are almost as twice as likely to develop major depression as the general population [53]. Chronic HIV-related distress may stem from a variety of reasons, ranging from the constant awareness of the presence of a lethal virus in the body [45], HIV-related physical symptoms and the necessity to adhere to daily treatment regimens [53] to struggling with various social problems arising from stigmatisation [54]. However, in more recent studies, this latter negative stressor, i.e. HIV-related stigma, has specifically been treated as the most important factor contributing to the constant presence of negative affectivity in this patient group. Particularly, internalised HIV-related stigma deteriorates the capacity to cope with the daily distress and causes long-term emotion dysregulation, which is additionally related to poor health outcomes [55].

Interestingly, poor affective well-being has been shown in many studies to be independent of social resources, i.e., high levels of depressive symptoms have been observed among PLWH with various levels of socioeconomic status and differential access to medical care [56]. In our study, the covariates of NA class membership were participants’ gender and CD4 count, both the CD4 baseline level and the CD4 change between the first and third assessment. On one hand, it is intriguing that the highest number of women (95% of all female participants) was found in the class with increasing NA, with decreased CD4 count as a significant covariate. In contrast, no female participants were found in the class with stable NA levels and the highest baseline CD4 count, where a further increase was observed. This finding may be considered in light of a consistent trend in the literature indicating lower levels of psychological well-being (PWB) among HIV-infected women in comparison to HIV-infected men [57, 58]. There are several factors that may be responsible for these results, such as unequal access to treatment and medical care in some countries for HIV-infected women [59], a high level of trauma and mental disorders among HIV-infected women [58], and, particularly, a much higher level of stigmatisation of HIV-infected women compared to HIV-infected men, which prevents many HIV-infected females from disclosing their HIV+ status and searching for support [60]. In this context, it is worth nothing that other than gender, sociodemographic variables did not differ between classes.

Additionally, the relationship between CD4 changes and NA trajectory is very interesting. More favourable trajectories (stable and decreasing) were related to CD4 count increases, whereas the less favourable trajectory, but the most frequent one, was related to CD4 count decreases over the course of the study. The literature on the role of immunological status as defined by CD4 count and well-being among PLWH provides a relatively coherent picture of a positive relationship; yet, this view is based mainly on cross-sectional studies. Several authors have observed a positive association between CD4 count and physical and emotional components of quality of life in this patient group [61, 62], and, particularly, a negative link between CD4 count and HIV-related depression [63, 64]. In the HIV epidemiology research study, Ickovicks et al. [65] found that depressive symptoms were significantly related to decreases in CD4 count and greater mortality rate among HIV-infected women, concluding that depressive symptoms may be linked to faster HIV progression when controlling for other socio-medical characteristics and substance use. Thus, it is likely that chronic or worsening NA may lead to a deterioration of immunological status, as defined by CD4 count, through a mediation of lowering treatment adherence, which was noted with respect to major depression in this patient group [20, 52]. Conversely, NA may also reflect a deterioration of immunological status itself as HIV infection progresses [52].

## Strengths and Limitations

This study has several strengths, including the longitudinal and theory-driven design, with three assessments of variables. While this time is probably crucial for the adaptation process [1], due to dynamics of affective states among people newly diagnosed with HIV, a different study design would be necessary (e.g. more and more frequent measurement points). Moreover, although we control for relatively vast numbers of socio-medical data, we did not have information about adherence to treatment or substance use among participants. Second, there were only three assessments that made a systematic analysis of other than linear trends in the trajectories impossible. In addition, relatively short time lags between consecutive assessments (6 months) may preclude obtaining a more thorough picture of the relationship between negative affect and CD4 count, especially that both variables were assessed only via self-report. Third, as we did not screen for clinical depression among participants, we are not able to specify how it may affect the results, particularly with regard to NA and CD4 count. It cannot be excluded that depression is a major explanatory variable here. Finally, a significantly lower number of women than men took part in this study, but this gender ratio can be regarded as typical for studies conducted among PLWH [66] and HIV epidemiology in general [67].

## Conclusion

Despite these limitations, our study adds to the HIV/AIDS literature by examining the possible trajectories of affect among PLWH and revealing the relationship between these trajectories and both gender and CD4 count changes. Specifically, these findings point to gender as a possible moderator in this process. This model calls for further investigation in prospective studies due to its potential clinical significance.
